# BDNF Haplotype and Personality Traits May Influence the Development of Alcohol Use Disorder in the Han Chinese Population

**DOI:** 10.1111/adb.70074

**Published:** 2025-08-06

**Authors:** Yi‐Hsin Wang, Yi‐Wei Yeh, Chun‐Long Lin, Shin‐Chang Kuo, Chun‐Yen Chen, Yu‐Chieh Huang, Ta‐Chuan Yeh, Chih‐Yun Huang, Kuo‐Hsing Ma, San‐Yuan Huang

**Affiliations:** ^1^ Department of General Medicine, Tri‐Service General Hospital National Defense Medical Center Taipei Taiwan (ROC); ^2^ Department of Psychiatry, Tri‐Service General Hospital National Defense Medical Center Taipei Taiwan (ROC); ^3^ Graduate Institute of Medical Sciences National Defense Medical Center Taipei Taiwan (ROC); ^4^ Department of Psychiatry, Hsinchu Branch Taoyuan Armed Forces General Hospital Hsinchu Taiwan (ROC); ^5^ Department of Anatomy and Biology National Defense Medical Center Taipei Taiwan (ROC); ^6^ Brain Research Center National Defense Medical Center Taipei Taiwan (ROC)

## Abstract

Brain‐derived neurotrophic factor (BDNF) and personality traits play a crucial role in the development of alcohol use disorder (AUD). However, the relationship between the *BDNF* gene, personality and AUD remains unclear. This study aimed to explore the role of BDNF gene variants, personality traits and impulsivity in both the presence of AUD and the age of onset of alcohol dependence. We also examined the interaction between BDNF gene variants and personality traits among individuals with AUD. Eleven polymorphisms encompassing the whole BDNF gene region were genotyped. The Tridimensional Personality Questionnaire (TPQ) and the Barratt Impulsiveness Scale‐11 (BIS‐11) assessed personality traits and impulsivity. Pearson's Chi‐square, HAPLOVIEW software, logistic regression, independent *t* tests and linear regression were conducted to investigate the associations between BDNF variants, personality traits and impulsivity in patients with AUD. Five‐hundred eighty‐five AUD patients and 546 normal controls (NC) were recruited for the study. Although no polymorphisms were significantly associated with AUD after Bonferroni correction in gender‐ or age‐stratified analyses, one specific haplotype block (TCA haplotype of rs1519480/rs6265/rs11030101) was related to AUD (*p* = 6.16 × 10 ^−5^ ). TPQ and BIS‐11 results revealed that AUD patients, particularly those with early‐onset AUD (EOAUD), exhibited significantly higher scores in Novelty Seeking (NS) and Harm Avoidance (HA) compared to NC. Additionally, the *BDNF* SNP rs6484320 was significantly associated with higher BIS‐11 scores in the AUD group. High HA, NS, impulsivity, and the BDNF gene's specific haplotype may influence AUD's development. The findings may provide new insights into the genetic and psychological factors contributing to AUD.

## Graphical abstract

Genetic and personality factors jointly shape alcohol use disorder (AUD). We identified a BDNF haplotype associated with AUD and found that higher novelty seeking, harm avoidance and impulsivity characterise patients, especially early‐onset cases. These findings provide insights into AUD vulnerability.

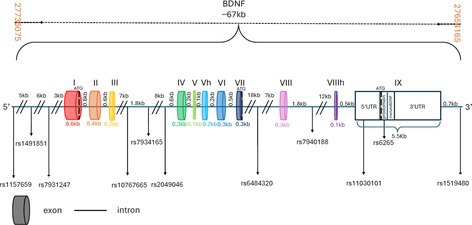

## 1 Introduction

Alcohol use disorder (AUD) poses a significant global health concern, affecting 3.7% of the world population and contributing to approximately 3 million deaths [1]. As a chronic mental disorder, AUD often originates from initial alcohol consumption driven by sensations of euphoria, relaxation or temporary stress and anxiety relief [2]. This positive reinforcement may evolve into negative reinforcement, which may lead to addiction [3]. AUD is a multifactorial syndrome influenced by diverse risk factors distributed across biological, psychological, social‐cultural levels and genetic factors [4]. Genetic factors play a substantial role in AUD development, with familial research indicating a high heritability rate for alcoholism (0.4–0.6) in both men and women [5, 6]. However, identifying susceptibility genes remains challenging due to study variations in design, subtypes, controls, gender bias and racial heterogeneity.

AUD is characterised as an addiction disease marked by the dysregulation of motivational brain circuits. The proposed mechanism for this addiction involves aberrations in learning and memory [7]. According to this theory, addictive substances enhance positive learning and memory related to their consumption while inhibiting awareness of the negative consequences associated with such behaviour [7]. In animal models, analyses reveal selective neuronal loss, dendritic simplification and reductions in synaptic complexity in specific brain regions [8]. Transitioning to evidence from human studies, heavy drinking affected brain regions, including the amygdala, ventral tegmental area and substantia nigra [9]. Clinical studies using magnetic resonance imaging (MRI) observe reduced surface area and volumes in the brain striatum of long‐term alcohol consumers with relapsing patterns. MRI scans reveal these structural changes, manifested as decreased grey matter density and volume reduction in the striatum [10].

Neurotrophins, which regulate various aspects of neural circuit development and structural changes, may play a crucial role in substance use disorder [11, 12]. The brain‐derived neurotrophic factor (*BDNF*) is the most prevalent neurotrophin in the brain. The mature *BDNF* protein is critical in regulating synapses, cognition and memory formation [13]. Animal models have suggested that alcohol intake initially increases BDNF levels as a protective mechanism, whereas chronic excessive alcohol consumption leads to BDNF dysregulation and reduced expression [14]. Human studies support these findings, showing significantly decreased plasma BDNF concentrations in abstinent alcohol use disorder patients compared to healthy controls [15]. According to the previous study, specific single‐nucleotide polymorphisms (SNPs) have been proven to be associated with changes in the expression of *BDNF* protein [16]. However, the relationship between genetic variations of *BDNF* and the development of AUD remains conflicted [17, 18].

In addition to biological factors, personality traits also influence the risk of AUD. Specifically, impulsive and sensation‐seeking traits have been empirically demonstrated to be crucial in sustaining binge drinking behaviours [19]. Genetic factors account for approximately one third to one half of interindividual differences in personality traits [20]. BDNF polymorphism has been demonstrated to be associated with anxiety traits [21]. Furthermore, Cloninger's typology of alcoholism posits that these personality traits may manifest differently across age groups in individuals with AUD [22]. Notably, BDNF research has shown that the age of first substance use moderates the difference in BDNF levels between drug users and healthy controls [23]. These findings collectively stimulate interest in exploring the relationship between age of onset, BDNF expression and personality traits in AUD.

An intricate interplay of genetic variants, personality traits and impulsiveness influences AUD. Our study aims to investigate *BDNF* variants in the development of AUD. Additionally, to achieve a more comprehensive understanding of AUD formation and the distinct phenotypic manifestations associated with different ages of onset, we explore the effects of personality traits and impulsiveness on the progression of AUD. Through this thorough analysis, we hope to contribute valuable insights into the intricate factors contributing to AUD and enhance our understanding of the underlying mechanisms.

## 2 Methods

### 2.1 Trial Design

This research was conducted at the Tri‐Service General Hospital (TSGH), a medical teaching hospital affiliated with the National Defense Medical Center in Taipei, Taiwan, in compliance with the 1994 Declaration of Helsinki and other ethical standards. Approval was obtained from the Institutional Review Board for the Protection of Human Subjects (TSGHIRB 107‐05‐011). All participants were unrelated Han Chinese individuals residing in Taiwan. To ensure the independence of our samples, we conducted family history interviews with each participant. Before participating, subjects were thoroughly informed about the study and provided their written consent. The cross‐sectional study investigated whether genetic variants (BDNF haplotypes), different personality traits and impulsivity were possible risk factors in the development of Taiwan Han Chinese AUD.

### 2.2 Sample and Recruitment

AUD subjects were recruited from the alcohol research and rehabilitation centres of TSGH in Taiwan between 1 January 2016 and 31 December 2023. General psychiatrists or psychologists made the initial diagnosis of AUD and subsequently confirmed it through detailed interviews conducted by professional addiction psychiatrists. These detailed clinical interviews used the modified Schedule for Affective Disorders and Schizophrenia‐Lifetime (SADS‐L) [24, 25] and diagnostic criteria from the Diagnostic and Statistical Manual of Mental Disorders, Fifth Edition (DSM‐5), to confirm AUD and assess for any co‐occurring mental illness. AUD participants with comorbid organic brain disorders, serious medical illnesses, major psychiatric illnesses or other substance use disorders were excluded (except nicotine use disorder [NUD] due to the high comorbidity between AUD and NUD). Healthy control subjects without personal or family history of psychiatric disorders were recruited from the general population in Taiwan. The same SADS‐L method and DSM‐5 criteria were used to verify their mental health status, including screening for other psychiatric disorders. Following standardised DSM‐5 guidelines, this meticulous diagnostic process ensured a well‐characterised sample for our study.

### 2.3 Assessment of Personality Traits and Impulsivity

The Chinese version of the complete Tridimensional Personality Questionnaire (TPQ) and Barratt Impulsiveness Scale (BIS‐11) was administered to evaluate personality traits and impulsivity in both AUD and normal control (NC) groups. The BIS11 contained three subscales: the attention impulsivity scale, which measured deficits in concentration and attention; the motor impulsivity scale, which evaluated fast reactions and restlessness; and the nonplanning impulsivity scale, which assessed planning and thinking capacity [26, 27]. In the Chinese version of the BIS‐11, the Cronbach *α* coefficient was 0.85, and the intraclass correlation coefficient was 0.68 among AUD participants in Taiwan, indicating that the Chinese version of the BIS‐11 is a reliable assessment tool. Due to the satisfactory reliability of Novelty Seeking (NS) and Harm Avoidance (HA) dimensions in the Taiwanese population, these two dimensions were included in the analysis, whereas the reward dependence dimension was omitted because of its low reliability [26, 28].

### 2.4 Selection and Genotyping of *BDNF* Variants

**FIGURE 1:**
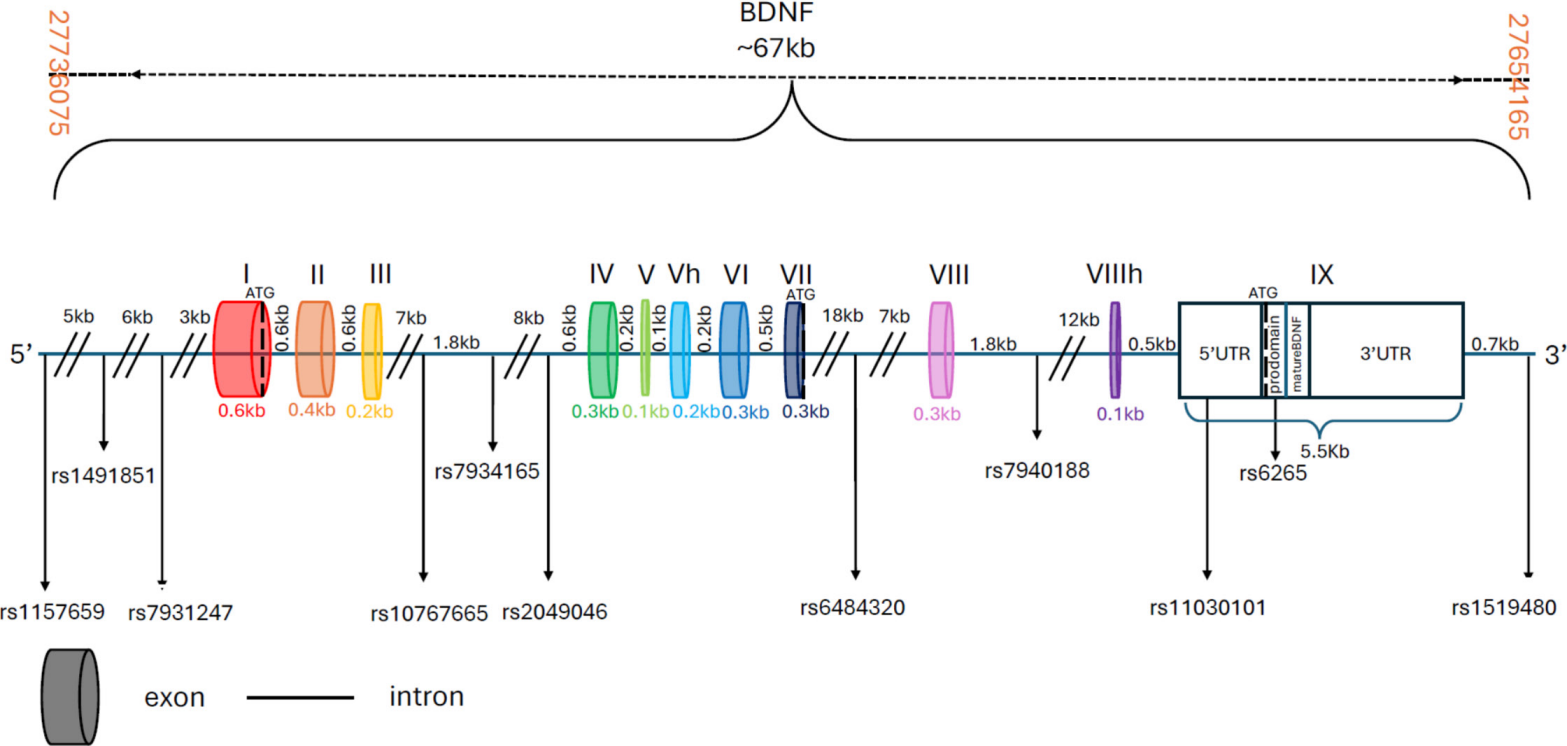
BDNF gene structure with the position of polymorphisms used in this study. By a random SNP selection method, 11 markers in the BDNF gene were selected.

The genetic SNPs were selected based on a thorough review of human BDNF polymorphisms listed in the NCBI SNP database (https://www.ncbi.nlm.nih.gov/variation/view/) and the 1000 Genomes Project database (https://www.internationalgenome.org/). We identified 11 SNPs within the BDNF gene and the minor allele frequencies (MAFs) > 0.1, spanning an 81‐kb region on chromosome 11P, including rs11576590, rs1491851 and rs7931247 in the promoter region; rs10767665, rs7934165 and rs2049046 in the intron between exons IV and III; rs6484320 in the intron between exons VII and VIII; rs7940188 in the intron between exons VIII and VIIIh; rs11030101 and rs6265 in exon IX; and rs1519480 in the 3′ end. All selected SNPs had minor allele frequencies greater than 0.1. As shown in Figure 1, the *BDNF* gene spans approximately 67 kb on chromosome 11p14.1, with these chosen polymorphisms distributed across various exons and introns. Figure 1 highlights the gene's structure, illustrating the exact positions of these SNPs along the *BDNF* gene, with specific attention to rs6265 in exon IX, a well‐known variant affecting the coding sequence (CDS).

After obtaining informed consent, genomic DNA was isolated from participants' peripheral blood leukocytes using a commercial kit (DNAzol; Invitrogen, Carlsbad, CA, United States). TaqMan assays (Thermo Fisher Scientific, made in the United States) with FAM and VIC dyes and the TagMan Universal Master Mix II, with UNG (Applied Biosystems by Thermo Fisher Scientific) were used in the genotyping PCR protocol. Details of the genotyping procedure are given below: (1) Extract high‐quality genomic DNA, and dilute DNA to 20 ng/μL; (2) reaction setup with 5‐μL TagMan genotyping Master Mix + 0.5 μL TaqMan assays (20X) + 1–2 μL genomic DNA (10 ng) and add ddH2O to reach a total of 10 μL for each reaction; (3) thermal cycle conditions: initial denaturation at 95°C for 10 min; then, enter the PCR cycling (40 cycles) including denaturation at 95°C for 15 s, and annealing/extension at 60°C for 1 min (data collection at this step) for each PCR cycle; (4) data collection and analysis: The Applied Biosystems StepOneTM software and StepOnePlusTM Real‐Time PCR Systems were used to read fluorescence and analyse allelic discrimination. To ensure genotyping reliability, a blind replication was conducted on 50 randomly selected samples, and all the SNPs were verified by RFLP and Sanger sequencing.

### 2.5 Data Analyses

All statistical analyses were conducted using IBM SPSS Statistics version 29.0 (IBM Corp., Armonk, NY, United States). Pearson's Chi‐square test compared allele and genotype frequencies between AUD patients and NC. We also applied Fisher's exact test when expected cell frequencies were below 5. Logistic regression was first used to evaluate associations between BDNF gene polymorphisms (as independent variables) and AUD (as dependent variables), adjusting for age and gender as covariates. We further stratified the analysis by the age of AUD onset into two groups: EOAUD (Early Onset of AUD, with onset age ≤ 25 years) and LOAUD (Late Onset of AUD, with onset age > 25 years) [29]. Haplotype analysis using HAPLOVIEW software (Version 4.2) examined linkage disequilibrium, haplotype frequencies, block structures and associations [30]. Additionally, we performed a permutation test for 1000 iterations to confirm the stability of the significance. Normality was first assessed; TPQ and BIS‐11 subscales that met normality were compared between AUD and controls with independent *t* tests, whereas nonnormal subscales were analysed using Mann–Whitney *U* tests. A linear regression analysis investigated associations between *BDNF* polymorphisms (as independent variables) and personality traits/impulsiveness (as dependent variables), adjusting for age, gender and nicotine use as covariates. For all analyses, significance was set at *p* < 0.05. Bonferroni correction was applied to adjust for multiple comparisons. With 11 SNPs tested, the significance threshold was set at *p* < 0.005 (0.05/11) after correction. Power analysis using G*POWER 3.1 software confirmed our sample size had high statistical power (> 0.90) to detect small to large effects.

## 3 Results

### 3.1 Demographic Data

Our study initially consisted of 1159 individuals, but 28 were excluded due to incomplete gene data. The final analysis included 1131 participants: 585 individuals with AUD and 546 NC. The mean age of the AUD group was 41.00 years (SD = 11.646), whereas the NC group had a mean age of 39.53 years (SD = 12.094). Independent *t* test showed no significant difference in age between the two groups (*p* = 0.053). Regarding gender distribution, the AUD group comprised 87.0% males and 13.0% females, whereas the NC group comprised 67.0% males and 33.0% females, a significant difference in gender distribution between the AUD and NC groups (*p* < 0.001). The severity scores of AUD patients based on DSM‐5 criteria averaged 8.3 ± 2.1 in this study, indicating predominantly severe AUD in our sample. We used the Visual Analogue Scale of craving to assess alcohol craving (0–10 scores). These patients had an average craving score of 7.2 ± 2.1, indicating high subjective craving intensity during the recruitment state. Regarding family history, 42.5% of AUD patients reported a third‐degree relative with problem drinking. Moreover, 88.1% of individuals in the AUD group reported nicotine use, whereas none of the participants in the NC group used nicotine, as nicotine use was an exclusion criterion for controls.

### 3.2 Relationship Between *BDNF* Gene Polymorphisms and AUD

Our analysis encompassed 11 *BDNF* gene SNPs, as detailed in Table 1. All BDNF SNPs were confirmed to be in Hardy–Weinberg (HW) equilibrium both in the AUD patients and NC groups (HW *p* value > 0.1), as assessed using the HAPLOVIEW software (Version 4.2). Most SNPs did not exhibit statistically significant differences in allele or genotype frequencies between AUD patients and NC. In our gender‐stratified analysis, we initially observed that rs11030101 demonstrated a significant association with AUD risk, specifically in the female subgroup (*p* = 0.013). However, after applying the Bonferroni correction for multiple comparisons, this association no longer reached statistical significance.

**TABLE 1:** Gene location, allele and genotype frequencies of the investigated *BDNF* gene polymorphisms among patients with AUD and control.

Variants	Location in BDNF	dbSNP reference Position	MAF		*p* ^a^	Allele^b^			Total NC (*n* = 546)			Total AUD (*n* = 585)			*p* ^d^
									Genotype, *n* (%)			Genotype, *n* (%)			
			ad	NC		1	2	Asian^c^	1/1	1/2	2/2	1/1	1/2	2/2	
rs1519480	3′ end	27654165	0.240	0.221	0.272	***C***	T	**C**	22 (4.0)	197 (36.1)	327 (59.9)	41 (7.0)	194 (34.0)	341 (59.0)	0.087
rs6265	Exon9	27658369	0.504	0.473	0.143	***C***	T	**T**	124 (22.7)	269 (49.3)	153 (28.0)	146 (25.0)	298 (50.9)	141 (24.1)	0.289
rs11030101	Exon9	27659197	0.260	0.250	0.592	***T***	A	**T**	27 (4.9)	219 (40.1)	300 (54.9)	45 (7.7)	214 (36.6)	326 (55.7)	0.117
rs7940188	Intron8	27672192	0.171	0.158	0.389	***C***	G	**C**	9 (1.6)	154 (28.2)	383 (70.1)	18 (3.1)	164 (28.0)	403 (68.9)	0.281
rs6484320	Intron7	27681641	0.513	0.489	0.258	***A***	T	**A**	128 (23.4)	278 (50.9)	140 (25.6)	151 (25.8)	298 (50.9)	136 (23.2)	0.521
rs2049046	Intron3	27702228	0.443	0.421	0.303	***A***	T	**A**	88 (16.1)	284 (52.0)	174 (31.9)	110 (18.8)	298 (50.9)	177 (30.3)	0.480
rs7934165	Intron3	27710436	0.442	0.430	0.582	***A***	G	**A**	94 (17.2)	282 (51.6)	170 (31.1)	109 (18.6)	299 (51.1)	177 (30.3)	0.818
rs10767665	Intron3	27712311	0.443	0.423	0.346	***A***	G	**A**	88 (16.1)	286 (52.4)	172 (31.5)	110 (18.8)	298 (50.9)	177 (30.3)	0.491
rs7931247	promotor	27725444	0.438	0.432	0.765	***C***	T	**C**	94 (17.2)	284 (52.0)	168 (30.8)	106 (18.1)	301 (51.5)	178 (30.4)	0.924
rs1491851	promotor	27731216	0.291	0.293	0.934	***C***	T	**C**	52 (9.5)	216 (39.6)	278 (50.9)	46 (7.9)	249 (42.6)	290 (49.6)	0.445
rs1157659	promotor	27736075	0.287	0.283	0.825	***G***	A	**G**	45 (8.2)	219 (40.1)	282 (51.6)	49 (8.4)	238 (40.7)	298 (50.9)	0.972

Variants	Male NC (*n* = 366)			Male AUD (*n* = 509)			*p* ^d^	Female NC (*n* = 180)			Female AUD (*n* = 76)			*p*
	Genotype, *n* (%)			Genotype, *n* (%)				Genotype, *n* (%)						
	1/1	1/2	2/2	1/1	1/2	2/2		1/1	1/2	2/2	1/1	1/2	2/2	
rs1519480	15 (4.1)	117 (32.0)	234 (63.9)	38 (7.5)	173 (34.0)	298 (58.5)	0.072	7 (3.9)	80 (44.4)	93 (51.7)	3 (3.9)	26 (34.2)	47 (61.8)	0.329^e^
rs6265	185 (23.2)	174 (47.5)	107 (29.2)	125 (24.6)	264 (51.9)	120 (23.6)	0.167	39 (21.7)	95 (52.8)	46 (25.6)	21 (27.6)	34 (44.7)	21 (27.6)	0.453^d^
rs11030101	22 (6.0)	150 (41.0)	194 (53.0)	36 (7.1)	190 (37.3)	283 (55.6)	0.507	5 (2.8)	69 (38.3)	106 (58.9)	9 (11.8)	24 (31.6)	43 (56.6)	0.013^d^
rs7940188	5 (1.4)	96 (26.1)	265 (72.4)	14 (12.8)	144 (28.3)	351 (69.0)	0.277	4 (2.2)	58 (32.2)	118 (65.6)	4 (5.3)	20 (26.3)	52 (68.4)	0.323^e^
rs6484320	89 (24.3)	178 (48.6)	99 (27.0)	126 (24.8)	265 (52.1)	118 (23.2)	0.408	39 (21.7)	100 (55.6)	41 (22.8)	25 (32.9)	33 (43.4)	18 (23.7)	0.121^d^
rs2049046	61 (16.7)	188 (51.4)	117 (32.0)	91 (17.9)	263 (51.7)	155 (30.5)	0.843	27 (15.0)	96 (53.3)	57 (31.7)	19 (25.0)	35 (46.1)	22 (28.9)	0.161^d^
rs7934165	63 (17.2)	187 (51.1)	116 (31.7)	90 (17.7)	264 (51.9)	155 (30.5)	0.924	31 (17.2)	95 (52.8)	54 (30.0)	19 (25.0)	35 (46.1)	22 (28.9)	0.341^d^
rs10767665	61 (16.7)	189 (51.6)	116 (31.7)	91 (17.9)	263 (51.7)	155 (30.5)	0.867	27 (15.0)	97 (53.9)	56 (31.1)	19 (25.0)	35 (46.1)	22 (28.9)	0.158^d^
rs7931247	65 (17.8)	185 (50.5)	116 (31.7)	88 (17.3)	266 (52.3)	155 (30.5)	0.881	29 (16.1)	99 (55.0)	52 (28.9)	18 (23.7)	35 (46.1)	23 (30.3)	0.284^d^
rs1491851	32 (8.7)	149 (40.7)	185 (50.5)	43 (8.4)	216 (42.4)	250 (49.1)	0.878	20 (11.1)	67 (37.2)	93 (51.7)	3 (3.9)	33 (43.4)	40 (52.6)	0.172^e^
rs1157659	27 (7.4)	149 (40.7)	190 (51.9)	46 (9.0)	205 (40.3)	258 (50.7)	0.678	18 (10.0)	70 (38.9)	92 (51.1)	3 (3.9)	33 (43.4)	40 (52.6)	0.279^e^

Abbreviations: AUD, alcohol use disorder; MAF, minor allele frequency; NC, normal controls.

^a^ Allele frequency in patients with AUD compared with the control group using the Pearson Chi‐square test.

^b^ Allele 1, which is italicised and bold, indicates the minor allele.

^**c**^ Reported MAFs in Asian populations were obtained from the NCBI dbSNP database (https://www.ncbi.nlm.nih.gov/snp/).

^d^ Genotype frequencies in patients with AUD or its subgroups compared with the controls using the Pearson Chi‐square test.

^e^ Genotype frequencies in patients with AUD or its subgroups compared with the controls using Fisher's exact test.

### 3.3 Relationship Between *BDNF* Gene Polymorphism and AUD in Age‐Stratified Subgroups

Separate logistic regression models were performed under a recessive genetic model to investigate the association between BDNF gene polymorphisms (as an independent variable) and AUD risk (as a dependent variable), controlling for age and gender. As shown in Table 2, in the group of total AUD, the rs1519480 (OR = 0.600, *p* = 0.067), rs11030101 (OR = 0.65, *p* = 0.097) and rs7940188 (OR = 0.463, *p* = 0.074 in total AUD; and OR = 0.340, *p* = 0.021 in LOAUD group) revealed that the major allele carriers have a protective trend against AUD. However, no significant finding was noted after Bonferroni correction for multiple comparisons.

**TABLE 2:** A logistic regression analysis of the BDNF gene polymorphisms as risk factors for alcohol use disorder, with correction for age and gender.

Variants	Variants (reference)	Total AUD (*n* = 585)			EOAUD (*n* = 212)			LOAUD (*n* = 373)		
		OR^b^	95% CI	*p* ^c^	OR^b^	95% CI	*p* ^d^	OR^b^	95% CI	*p* ^e^
rs1519480	(C/C)^a^	1			1			1		
	C/T + T/T	0.600	0.347–1.037	0.067	0.674	0.326–1.393	0.287	0.561	0.302–1.042	0.067
rs6265	(C/C)^a^	1			1			1		
	C/T + T/T	0.857	0.644–1.141	0.291	0.803	0.548–1.176	0.259	0.874	0.627–1.220	0.429
rs11030101	(T/T)^a^	1			1			1		
	T/A + A/A	0.650	0.391–1.081	0.097	0.732	0.372–1.444	0.368	0.632	0.356–1.120	0.116
rs7940188	(C/C)^a^	1			1			1		
	C/G + G/G	0.463	0.199–1.078	0.074	0.837	0.240–2.919	0.781	0.340	0.136–0.851	0.021
rs6484320	(A/A)^a^	1			1			1		
	A/T + T/T	0.834	0.629–1.107	0.209	0.804	0.550–1.175	0.259	0.875	0.630–1.213	0.875
rs2049046	(A/A)^a^	1			1			1		
	A/T + T/T	0.805	0.584–1.109	0.185	0.854	0.553–1.320	0.478	0.788	0.546–1.139	0.205
rs7934165	(A/A)^a^	1			1			1		
	A/G + G/G	0.869	0.632–1.193	0.384	0.907	0.589–1.397	0.658	0.855	0.594–1.232	0.402
rs10767665	(A/A)^a^	1			1			1		
	A/G + G/G	0.802	0.582–1.105	0.178	0.870	0.563–1.344	0.530	0.771	0.534–1.114	0.166
rs7931247	(C/C)^a^	1			1			1		
	C/T + T/T	0.919	0.669–1.263	0.604	0.987	0.636–1.530	0.952	0.905	0.629–1.303	0.591
rs1491851	(C/C)^a^	1			1			1		
	C/T + T/T	1.198	0.777–1.845	0.413	1.287	0.700–2.367	0.417	1.107	0.671–1.828	0.691
rs1157659	(G/G)^a^	1			1			1		
	G/A + A/A	0.946	0.608–1.470	0.804	0.972	0.534–1.769	0.926	0.887	0.532–1.480	0.647

Abbreviations: CI, confidence interval; EOAUD, early onset of AUD, with onset before age 25 years (≤ 25 years old); LOAUD, late onset of AUD, with onset after age 25 years (> 25 years old); OR, odds ratio.

^a^ Genotype within parenthesis indicates the reference group of genotypes.

^b^ Odds ratio is given with 95% confidence intervals (95% CI) after a logistic regression analysis.

^c^ Healthy controls (*n* = 537) versus patients with AUD (*n* = 585).

^d^ Healthy controls (*n* = 537) versus patients with EOAUD (*n* = 212).

^e^ Healthy controls (*n* = 537) versus patients with LOAUD (*n* = 373).

### 3.4 Relationship Between BDNF Gene Haplotype Blocks and AUD

**FIGURE 2:**
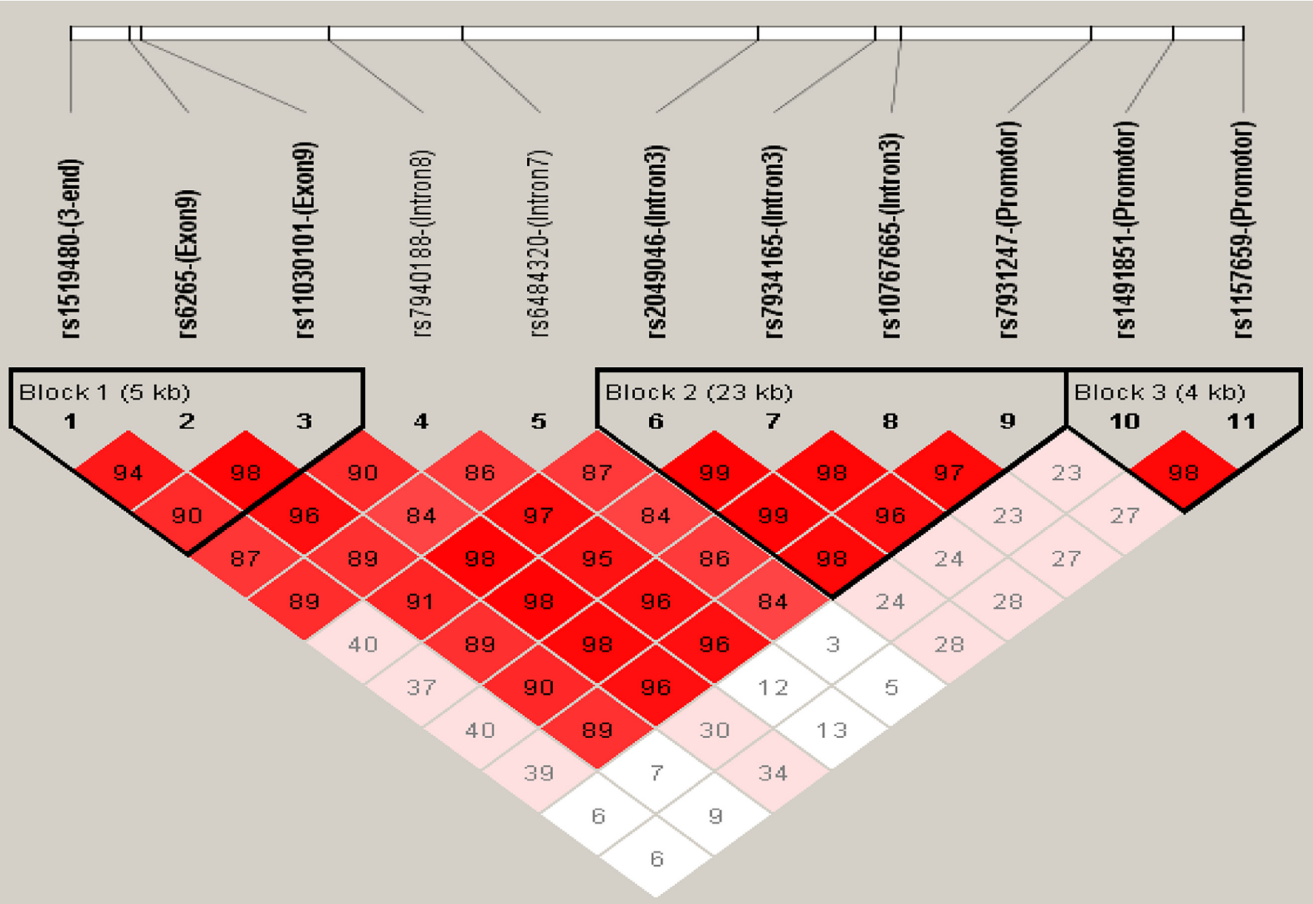
Linkage disequilibrium (LD) plot of single nucleotide polymorphisms (SNPs) in the gene region of interest. Three blocks were identified: Block 1 (6 kb), Block 2 (23 kb) and Block 3 (4.5 kb). SNP IDs are displayed at the top of the figure.

The haplotype analysis was used to better understand the *BDNF* variant's relationship with AUD. Figure 2 illustrates three haplotype blocks and the linkage disequilibrium (LD) structure of the studied *BDNF* gene. In the total AUD subjects, haplotype analysis revealed several associations, as detailed in Table 3. Within haplotype Block 1, the TTA haplotype of rs1519480/rs6265/rs11030101, the most common in both groups (0.524 in NC and 0.483 in total AUD patients), demonstrated a borderline protective effect against AUD (*p* = 0.050) before Bonferroni correction. Additionally, the relatively low‐frequency TCA haplotype of rs1519480/rs6265/rs11030101 showed significant associations with AUD risk in the total AUD group (*p* = 6.16 × 10^−5^), EOAUD group (*p* = 0.002) and LOAUD group (*p* = 4.23 × 10^−5^). Moreover, within haplotype Block 2, the TGGC haplotype of rs2049046/rs7934165/rs10767665/rs7931247 is significantly associated with total AUD (*p* = 0.009) and LOAUD (*p* = 0.006). The TAGT haplotype of rs2049046/rs7934165/rs10767665/rs7931247 is also significantly related to total AUD (*p* = 0.009). However, only the TCA haplotype of rs1519480/rs6265/rs11030101 remained significant after the Bonferroni correction (*p* < 0.002). In contrast, no associations between haplotypes and AUD were observed in Block 3.

**TABLE 3:** Haplotype analysis of *BNDF* gene in patients with AUD (*n* = 585) and controls (*n* = 546).

Haplotype Block 1			Frequency			Frequency		Frequency	
rs1519480	rs6265	rs11030101	Total NC	Total AUD	*p* ^a^	EOAUD	*p* ^b^	LOAUD	*p* ^c^
T	T	A	0.524	0.483	0.050	0.470	0.061	0.490	0.160
T	C	T	0.248	0.248	0.993	0.249	0.984	0.247	0.962
C	C	A	0.220	0.218	0.950	0.231	0.622	0.211	0.674
T	C	A	0.006	0.028	6.16 × 10^−5^	0.024	0.002	0.030	4.23 × 10^−5^

Haplotype Block 2				Frequency			Frequency		Frequency	
rs2049046	rs7934165	rs10767665	rs7931247	Total NC	Total AUD	*p* ^a^	EOAUD	*p* ^b^	LOAUD	*p* ^c^
T	G	G	T	0.554	0.552	0.924	0.547	0.809	0.556	0.957
A	A	A	C	0.416	0.431	0.493	0.436	0.484	0.429	0.623
T	G	G	C	0.010	0.002	0.009	0.005	0.310	0.001	0.006
T	A	G	T	0.010	0.002	0.009	0.001	0.038	0.001	0.033
A	A	A	T	0.003	0.008	0.107	0.012	0.030	0.004	0.378

Haplotype Block 3		frequency			frequency		frequency	
rs1491851	rs1157659	Total NC	Total AUD	*p* ^a^	EOAUD	*p* ^b^	LOAUD	*p* ^c^
T	A	0.705	0.703	0.929	0.686	0.473	0.713	0.710
C	G	0.281	0.282	0.962	0.295	0.597	0.275	0.766
C	A	0.012	0.009	0.563	0.009	0.683	0.009	0.609

Abbreviations: EOAUD, early onset of AUD, with onset before age 25 years (≤ 25 years old); LOAUD, late onset of AUD, with onset after age 25 years (> 25 years old).

^a^ Healthy controls (*n* = 537) versus patients with AUD (*n* = 585).

^b^ Healthy controls (*n* = 537) versus patients with EOAUD (*n* = 212).

^c^ Healthy controls (*n* = 537) versus patients with LOAUD (*n* = 373).

### 3.5 Difference in Personality Traits and Impulsiveness Between AUD and NC

Genetic association studies for AUD exhibit variability that may be influenced by both genetic and environmental factors, including personality traits. It is recognized that specific personality characteristics can affect the course and outcome of AUD, potentially reflecting a genetic susceptibility to AUD. Our findings support this concept, as AUD patients demonstrated elevated scores in NS and HA compared to NC. As shown in Table 4, in an assessment utilizing the Chinese versions of the TPQ and BIS‐11, 196 AUD patients and 182 NC participated in the BIS‐11 evaluation, whereas 283 AUD patients and 279 NC completed the TPQ assessment. For BIS‐11, NC scored 64.19 ± 8.00, whereas AUD patients scored 71.68 ± 8.65. For TPQ, NC had NS scores of 13.36 ± 4.51 and HA scores of 10.48 ± 5.36, whereas AUD patients scored 16.61 ± 4.78 and 16.26 ± 5.94, respectively. Using independent *t* tests for normally distributed measures (NS) and Mann–Whitney U tests for nonnormal distributions (HA, BIS‐11 total scores and all subscales), we found that BIS‐11 total scores and all subscales (BIS nonplan, BIS motor and BIS attention), as well as TPQ NS and HA scores, were significantly higher in the AUD group compared to NC (all *p* < 1 × 10^−6^). We then categorised AUD patients into EOAUD and LOAUD. As highlighted in Table 4, the EOAUD and AUD with onset > 25 groups maintained significantly higher scores than NC across all measures (BIS‐11 total and subscales, TPQ NS and HA; all *p* < 1 × 10^−6^). When comparing EOAUD to LOAUD, only NS scores showed a significant difference, with EOAUD patients exhibiting higher scores (18.35 ± 5.07 vs. 15.88 ± 4.47, *p* < 0.001). No significant differences were observed between EOAUD and LOAUD for BIS‐11 total score, BIS‐11 subscales or HA scores (all *p* > 0.05).

**TABLE 4:** Revision: Specific personality traits and impulsiveness between healthy controls and patients with AUD subgroup.

Scores	BISnoplan	*p* ^e^	BISmotor	*p* ^*e*^	BISattention	*p* ^*e*^	BISsum	*p* ^*e*^
Groups								
Healthy controls (*N* = 182)	24.46 ± 3.34		22.84 ± 3.87		16.90 ± 2.67		64.19 ± 8.00	
Total AUD (*N* = 196)	29.04 ± 5.09	1 × 10^–6^ ^a^	23.91 ± 4.90	0.104^a^	18.73 ± 3.81	< 1 × 10^–6^ ^a^	71.68 ± 8.65	< 1 × 10^–6^ ^a^
EOAUD (*N* = 57)	28.93 ± 5.51	< 1 × 10^–6^ ^b^	24.63 ± 6.35	0.257^b^	19.14 ± 4.07	< 1 × 10^–4^ ^b^	72.70 ± 9.99	< 1 × 10^–6^ ^b^
LOAUD (*N* = 139)	29.09 ± 4.93	< 1 × 10^–6^ ^c^	23.61 ± 4.15	0.142^c^	18.57 ± 3.70	< 1 × 10^–3^ ^c^	71.27 ± 8.04	< 1 × 10^–6^ ^c^
EOAUD versus LOAUD	0.879^d^		0.758^d^		0.123^d^		0.183^d^	

Scores	Novelty seeking score	*p* ^f^	Harm avoidance score	*p* ^*e*^
Groups				
Healthy controls (*N* = 279)	13.36 ± 4.51		10.48 ± 5.36	
Total AUD (*N* = 283)	16.61 ± 4.78	< 1 × 10^–6^ ^a^	16.26 ± 5.94	< 1 × 10^–6^ ^a^
EOAUD (*N* = 85)	18.35 ± 5.07	< 1 × 10^–6^ ^b^	16.95 ± 5.95	< 1 × 10^–6^ ^b^
LOAUD (*N* = 198)	15.88 ± 4.47	< 1 × 10^–6^ ^c^	15.96 ± 5.92	< 1 × 10^–6^ ^c^
EOAUD versus LOAUD	< 1 × 10^–3^ ^d^		0.187^d^	

Abbreviations: BIS, Barratt Impulsiveness Scale; EOAUD, early onset of AUD, with onset before age 25 years (≤ 25 years old); LOAUD, late onset of AUD, with onset after age 25 years (> 25 years old).

^a^ Patient with AUD versus healthy controls.

^b^ Patient with EOAUD versus Healthy controls.

^c^ Patient with LOAUD versus Healthy controls.

^d^ Patients with EOAUD versus Patients with LOAUD.

^e^ AUD (or their subgroups) and healthy controls were examined with Mann–Whitney *U* tests.

^f^ AUD (or their subgroups) and healthy controls were examined with independent‐samples *t* tests.

### 3.6 Relationship Between BDNF Gene Polymorphism, Personality Traits and Impulsivity

Separate linear regression models were performed under the dominant model to explore the relationship between BDNF gene SNPs polymorphisms (independent variable) and TPQ and BIS‐11 scores (dependent variable), controlling for age, gender and nicotine use. In AUD patients, our initial analysis revealed several nominally significant associations (Table 5). For the HA score, rs11030101 (*p* = 0.036), rs2049046 (*p* = 0.011), rs7934165 (*p* = 0.018), rs10767665 (*p* = 0.018) and rs7931247 (*p* = 0.036) showed potential associations. In the BIS nonplan subscale, rs1519480 (*p* = 0.047) and rs6484320 (*p* = 0.014) appeared significant. For the BIS motor subscale, rs6484320 (*p* = 0.026) showed a nominal association. Lastly, in the BIS sum score, rs1519480 (*p* = 0.034), rs6265 (*p* = 0.017), rs7940188 (*p* = 0.016) and rs6484320 (*p* < 0.001) initially demonstrated significant relationships. After applying the Bonferroni correction for multiple comparisons, only the association between rs6484320 and the BIS sum score in the AUD group remained statistically significant. We also performed similar analyses in the control group and the combined total group (AUD patients and NC). No significant associations were found between *BDNF* gene SNPs and personality traits or impulsive measures, even before Bonferroni correction in the NC group and combined total group analyses (Table S1 for whole samples and Table S2 for the NC group)

**TABLE 5:** Association of BDNF gene polymorphisms with specific personality traits and impulsiveness in AUD patients using dominant model linear regression, adjusting for age, gender and nicotine use disorder.

Variants	NS score (*n* = 283)				HA score (*n* = 283)				BIS nonplan (*n* = 196)			
	beta ^a^	95% CI	*p* ^b^	Partial *η* ^2^	beta ^a^	95% CI	*p* ^b^	Partial *η* ^2^	beta ^a^	95% CI	*p* ^b^	Partial *η* ^2^
rs1519480	0.055	−0.055 ~ 0.164	0.326	0.003	0.038	−0.078 ~ 0.154	0.516	0.002	−0.142	−0.282 ~ −0.002	0.047	0.02
rs6265	0.033	−0.076 ~ 0.143	0.55	0.001	−0.082	−0.198 ~ 0.034	0.164	0.007	−0.139	−0.279 ~ 0.001	0.051	0.02
rs11030101	0.009	−0.100 ~ 0.118	0.869	< 0.001	−0.122	−0.237 ~ −0.008	0.036	0.016	−0.005	−0.147 ~ 0.137	0.941	< 0.001
rs7940188	0.004	−0.106 ~ 0.114	0.945	< 0.001	−0.02	−0.137 ~ 0.096	0.729	0.001	−0.126	−0.266 ~ 0.014	0.077	0.016
rs6484320	−0.030	−0.139 ~ 0.079	0.588	0.001	−0.076	−0.191 ~ 0.039	0.195	0.006	−0.174	−0.313 ~ −0.035	0.014	0.031
rs2049046	0.004	−0.106 ~ 0.113	0.949	< 0.001	−0.149	−0.263 ~ −0.034	0.011	0.023	−0.115	−0.256 ~ 0.026	0.109	0.013
rs7934165	0.001	−0.109 ~ 0.110	0.995	< 0.001	−0.139	−0.254 ~ −0.024	0.018	0.02	−0.109	−0.251 ~ 0.032	0.128	0.012
rs10767665	0.001	−0.109 ~ 0.110	0.995	< 0.001	−0.139	−0.254 ~ −0.024	0.018	0.02	−0.109	−0.251 ~ 0.032	0.128	0.012
rs7931247	−0.002	−0.111 ~ 0.108	0.973	< 0.001	−0.123	−0.238 ~ −0.008	0.036	0.016	−0.106	−0.247 ~ 0.035	0.138	0.011
rs1491851	−0.006	−0.115 ~ 0.103	0.916	< 0.001	−0.073	−0.188 ~ 0.042	0.215	0.006	0.105	−0.037 ~ 0.246	0.145	0.011
rs1157659	0.003	−0.106 ~ 0.112	0.961	< 0.001	−0.067	−0.182 ~ 0.048	0.253	0.005	0.116	−0.025 ~ 0.257	0.107	0.014

Variants	BIS motor (*n* = 378)				BIS attention (*n* = 378)				BIS sum (*n* = 378)			
	beta ^a^	95% CI	*p* ^b^	partial *η* ^2^	beta ^a^	95% CI	*p* ^b^	partial *η* ^2^	beta ^a^	95% CI	*p* ^b^	partial *η* ^2^
rs1519480	−0.08	−2.219 ~ 0.614	0.265	0.006	−0.056	−0.199 ~ 0.087	0.44	0.003	−0.154	−0.295 ~ −0.012	0.034	0.023
rs6265	−0.092	−2.650 ~ 0.553	0.198	0.009	−0.088	−0.230 ~ 0.055	0.226	0.008	−0.173	−0.314 ~ −0.032	0.017	0.03
rs11030101	−0.043	−1.821 ~ 0.971	0.549	0.002	0.025	−0.118 ~ 0.169	0.729	0.001	−0.016	−0.160 ~ 0.127	0.821	< 0.001
rs7940188	−0.078	−2.219 ~ 0.636	0.275	0.006	−0.127	−0.268 ~ 0.015	0.079	0.016	−0.174	−0.315 ~ −0.033	0.016	0.03
rs6484320	−0.159	−3.555 ~ −0.232	0.026	0.026	−0.112	−0.254 ~ 0.030	0.121	0.013	−0.242	−0.381 ~ −0.103	< 0.001	0.058
rs2049046	−0.067	−2.302 ~ 0.820	0.35	0.005	−0.039	−0.183 ~ 0.104	0.588	0.002	−0.123	−0.266 ~ 0.020	0.091	0.015
rs7934165	−0.067	−2.310 ~ 0.830	0.354	0.005	−0.049	−0.192 ~ 0.095	0.505	0.002	−0.124	−0.267 ~ 0.019	0.09	0.015
rs10767665	−0.067	−2.310 ~ 0.830	0.354	0.005	−0.049	−0.192 ~ 0.095	0.505	0.002	−0.124	−0.267 ~ 0.019	0.09	0.015
rs7931247	−0.071	−2.363 ~ 0.792	0.327	0.005	−0.064	−0.207 ~ 0.079	0.38	0.004	−0.131	−0.273 ~ 0.012	0.072	0.017
rs1491851	−0.035	−1.730 ~ 1.052	0.631	0.001	−0.024	−0.168 ~ 0.120	0.743	0.001	0.032	−0.113 ~ 0.176	0.666	0.001
rs1157659	−0.023	−1.614 ~ 1.168	0.752	0.001	−0.013	−0.157 ~ 0.130	0.855	< 0.001	0.049	−0.094 ~ 0.193	0.499	0.002

Abbreviations: BIS, Barratt Impulsiveness Scale; HA, harm avoidance; NS, novelty seeking; partial *η* ^2^, partial eta squared.

^a^ Standardized beta coefficient, representing the mean difference in outcome between minor allele carriers and the reference genotype group.

^b^ The AUD patients with the reference group genotype versus the AUD patients with variant carriers.

## 4 Discussion

Previous studies have shown that *BDNF* gene SNPs are associated with various psychiatric disorders, including major depressive disorder, bipolar disorder, schizophrenia and even suicidal behaviour [31, 32]. However, in the domain of substance use disorders, studies have predominantly focused on the functional SNP rs6265, yielding inconsistent conclusions [17, 18]. In our analysis, rs6265 showed no significant association with AUD. In contrast, rs11030101 showed initial significance in the female group, though this did not persist after Bonferroni correction. Our study employed logistic regression to adjust for age and gender, and rs1519480‐T carrier, rs11030101‐A carrier and rs7940188‐G carrier were observed to have a borderline protective trend against AUD but no statistical significance. We also examined the association across different onset age groups. Previous research had established that EOAUD had stronger genetic components and familial associations. However, our findings presented a reversed pattern, with rs7940188 showing significance in LOAUD before the Bonferroni correction. Several factors may account for these differences. First, ethnic differences may play a crucial role. The minor allele frequency of rs7940188‐C was approximately 15.8% in our Han Chinese controls, considerably higher than the 13% found in the multiethnic population study [33]. A similar difference was also noted in rs6265‐C, with 47.3% in our data compared to 81.9% reported in the Caucasian population [18]. Our previous studies also identified a gene correlated with LOAUD, suggesting potential population‐specific genetic mechanisms in AUD development in Han Chinese [34]. Second, genetic factors may influence specific personality traits, potentially leading to LOAUD. Previous research has shown that neuropeptide Y, which is linked to anxiety levels, is associated with LOAUD [35]. This finding aligns with our current understanding of the psychological profile in LOAUD. BDNF may also play a role in personality characteristics and subsequently influence LOAUD risk, whereas specific personality‐related studies for rs7940188 are currently lacking. Third, the functional impact of BDNF polymorphisms may manifest through age‐related decline in neurological function, as evidenced by MRS studies showing reduced NAA levels with age [36]. This suggests that the genetic influence becomes more pronounced with advancing age, potentially contributing to the development of AUD in later life. Further studies incorporating both genetic and neuroimaging data are needed to validate these hypotheses and elucidate the mechanisms underlying the interaction between BDNF variants and age‐related changes in AUD development.

We analysed haplotypes better to understand the association between BDNF SNPs and AUD. We identified that the *BDNF* gene rs1519480/rs6265/rs11030101 TCA haplotype was significantly more frequent in the AUD group compared to the control group (*p* = 6.16 × 10^−5^), a finding noted in both EOAUD and LOAUD subgroups. These results suggest two key findings. First, the associations that were initially significant only before Bonferroni correction became more pronounced in the haplotype analysis, with similar observations across different subgroups. This finding aligns with a previous study on rs6265 and rs11030101, which reported no significant association at the individual SNP level but identified the rs11030101/rs2030324/rs6265 AAC haplotype as a significant risk factor for schizophrenia [37]. Second, the TCA and TTA haplotypes of 1 519 480/rs6265/rs11030101, which differ only in the rs6265 allele, exhibited a striking reversal in their association with AUD risk. Previous research on these loci has yielded several findings. Neuroimaging studies have shown that rs1519480 is associated with decreased levels of N‐acetyl‐aspartate, which may indicate neural integrity. In clinical studies, rs1519480 has been implicated in obsessive‐compulsive disorder and recovery from traumatic brain injury [38, 39]. Regarding rs11030101, it has been associated with depression and schizophrenia in previous studies [37, 40]. However, to the best of our knowledge, neither rs1519480 nor rs11030101 has been previously linked to substance use disorders, and research elucidating their biological mechanisms remains limited. The *T* allele of rs6265, which encodes methionine (Met), has been consistently associated with decreased BDNF protein function and secretion [16, 41]. Also, it may decrease hippocampal volume and reduce grey matter volume in Met carriers [42]. However, the *C* allele of rs6265 may act as a risk factor in our AUD patients (TCA haplotypes of 1 519 480/rs6265/rs11030101; Table 3). Similar results have been observed in previous studies on schizophrenia and obsessive‐compulsive disorder [37, 38]. Interestingly, a study of OCD patients found a significant association of rs1519480 with OCD, where the same rs6265 allele conferred either protection or risk depending on which rs1519480 variant it was paired with in haplotype analysis [38]. In our study, this rs6265‐C allele transition from a protective to a risk factor is observed only when this C allele is in conjunction with rs1519480‐T and rs11030101‐A, which may underscore the critical role of rs6265 in modulating AUD susceptibility. Notably, this effect was not apparent in the individual SNP analysis, highlighting the importance of haplotype‐based approaches in uncovering complex genetic associations.

Addiction is a multifactorial disease influenced by a complex interplay of factors extending beyond genetics, encompassing environmental influences and personality traits. Among these factors, personality structure also plays a particularly crucial role in the development and maintenance of AUD. We examined personality traits using the TPQ and BIS‐11 to investigate this aspect. Our analysis of the TPQ revealed significant relationships that align with patients with heroin dependence [26]. We observed marked differences in NS and HA scores between AUD patients and NC. Regarding impulsivity, our BIS‐11 findings are consistent with previous research that has consistently shown higher impulsivity in substance users [43], including alcohol users [44]. Our data demonstrate significantly elevated BIS‐11 scores across all subscales (nonplan, motor and attention) in AUD patients compared to NC (all *p* < 1 × 10^−6^). Our study further extends these findings by stratifying AUD patients based on age of onset. Previous literature has not extensively explored this age‐based stratification of personality traits and impulsivity. In addition to comparing AUD patients with NC, we conducted a detailed comparison between EOAUD and LOAUD. We found that EOAUD patients exhibited significantly higher NS scores than LOAUD (Table 4). This distinction aligns with and further supports the Type 1/Type 2 alcoholism hypothesis proposed by Cloninger [22].

Building upon these findings, we further explored the potential relationship between the identified *BDNF* SNPs and personality traits, including impulsivity, which previous studies have suggested may mediate the genetic influence on AUD development [45, 46]. In our research, we identified that rs11030101, rs2049046, rs7934165, rs10767665 and rs7931247 showed a significant effect on HA scores. Although these associations did not persist after Bonferroni correction, it is noteworthy that most of these SNPs are located within haplotype block 2. In addition, rs6484320 was significantly associated with the BIS sum score (*p* < 0.001) after Bonferroni correction. In previous animal studies, rats exhibiting higher impulsivity were observed to have significantly reduced *BDNF* mRNA in the dorsal hippocampus, suggesting a potential link between *BDNF* and impulsivity [47]. However, fundamental studies on the biological function of rs6484320 are lacking. In clinical research, rs6484320 has been associated with nicotine dependence in the specific haplotype pattern [48]. These results suggest that specific *BDNF* variants might influence personality traits or behavioural tendencies that could, in turn, affect vulnerability to AUD or its clinical presentation.

Despite the substantial sample size, some subgroup analyses and rare haplotypes may have been underpowered. The focus on Han Chinese individuals in Taiwan may limit the generalisability of findings to other ethnic populations. Although we excluded major psychiatric illnesses and other substance use disorders to reduce confounding factors, NUD was still included in our sample to maintain an adequate sample size for statistical analysis (and reflect the real‐world conditions of AUD patients), and the income issue was not used as a covariate. The significant gender imbalance in the AUD group (87% male) could potentially mask or exaggerate gender‐specific effects, particularly in subgroup analyses, despite being accounted for as a covariate in the main analysis. Moreover, due to methodological limitations, the haplotype analysis results were not adjusted for age and gender. This lack of adjustment in the haplotype analysis may limit our ability to accurately assess the influence of these demographic factors on the observed genetic associations. The age categorizations used for early‐onset (≤ 25 years) and late‐onset (> 25 years) AUD, although based on previous research, are somewhat arbitrary. It is crucial to acknowledge that the definition of EOAUD has varied across previous studies [49, 50]. In our investigation, we adopted 25 years as the threshold, based on Cloninger's classification of Types 1 and 2 alcoholism [22] and consistency with previous research that also used this age cutoff [29, 46]. However, this lack of standardised age thresholds in the field makes direct comparisons with other studies challenging and could contribute to inconsistencies in findings.

## 5 Conclusion

The specific TCA haplotype of rs1519480/rs6265/rs11030101 block was significantly overrepresented in the AUD group compared to controls, which suggests that the *BDNF* gene may play some role in the genetic loading of the development of AUD. The novelty‐seeking, harm‐avoidance and impulsivity scores are higher in AUD patients than in healthy controls, and higher novelty‐seeking scores are associated with early‐onset AUD than with late‐onset AUD. These results suggest that specific personality and impulsiveness may influence the development of AUD, and high novelty seeking may play a factor in young adults' heavy drinking. Our study also uncovered a significant association between the *BDNF* rs6484320 polymorphism and the BIS‐11 sum score in AUD patients. This result may suggest a potential gene–behaviour interaction specific to AUD. The study may provide valuable insights into the complex interplay between *BDNF* gene polymorphisms, impulsiveness, personality traits and the development of AUD across different age groups. Although the haplotype and specific personality approaches may reveal potential genetic influences, the heterogeneity of AUD requires further research.

## Author Contributions

S.Y.H. led the conceptualisation and overall study design and contributed to supervision and writing – review and editing. C.Y.H., T.C.Y., and S.C.K. carried out investigation and data curation through genotyping and data acquisition. Y.H.W., C.L.L., S.C.K., and C.Y.C. conducted formal analysis and data curation for data interpretation. K.S.M., S.Y.H., and Y.W.Y. supported interpretation and review and editing. Y.H.W. wrote the original draft. All authors critically reviewed the manuscript and approved the final version for publication.

## Ethics Statement

Approval was obtained from the Institutional Review Board for the Protection of Human Subjects (TSGHIRB 107‐05‐011).

## Consent

Participants provided voluntary informed consent to participate.

## Conflicts of Interest

The authors declare no conflicts of interest.

## Supporting information


**Table S1** Association of BDNF gene polymorphisms with specific personality traits and impulsiveness in the total sample using dominant model linear regression, adjusting for age, gender, and alcohol use disorder.
**Table S2** Association of BDNF gene polymorphisms with specific personality traits and impulsiveness in the control sample using dominant model linear regression, adjusting for age and gender.

## Data Availability

Data can be made available via data‐sharing agreements by request.
