# Supplementary material for: BDNF Haplotype and Personality Traits May Influence the Development of Alcohol Use Disorder in the Han Chinese Population
Source: Addict Biol. 2025 Aug 6;30(8):e70074. doi: 10.1111/adb.70074 (PMC12327066; doi:10.1111/adb.70074)
Supplement: Supplementary file 1 — Table S1 Association of BDNF gene polymorphisms with specific personality traits and impulsiveness in the total sample using dominant model linear regression, adjusting for age, gender, and alcohol use disorder. Table S2 Association of BDNF gene polymorphisms with specific personality traits and impulsiveness in the control sample using dominant model linear regression, adjusting for age and gender. [file ADB-30-e70074-s001.pdf]

Supplementary Table 1. Association of BDNF gene polymorphisms with specific personality traits and impulsiveness in the total sample using dominant model linear regression, adjusting for age, gender, and alcohol use disorder

Variants	NS score (n = 562)				HA score (n = 562)				BIS nonplan (n = 378)			
	beta [a]	95% CI	*p* [b]	partial η^2	beta [a]	95% CI	*p* [b]	partial η^2	beta [a]	95% CI	*p* [b]	partial η^2
rs1519480	0.003	-0.071 ~ 0.077	0.934	<0.001	-0.005	-0.079 ~ 0.068	0.892	<0.001	-0.038	-0.128 ~ 0.053	0.411	0.002
rs6265	-0.028	-0.102 ~ 0.046	0.451	0.001	-0.046	-0.120 ~ 0.027	0.216	0.003	-0.045	-0.136 ~ 0.045	0.326	0.003
rs11030101	-0.005	-0.079 ~ 0.069	0.899	<0.001	-0.037	-0.110 ~ 0.037	0.327	0.002	-0.019	-0.109 ~ 0.071	0.681	<0.001
rs7940188	-0.018	-0.092 ~ 0.056	0.631	<0.001	-0.033	-0.107 ~ 0.040	0.372	0.001	-0.042	-0.132 ~ 0.049	0.366	0.002
rs6484320	-0.066	-0.140 ~ 0.007	0.078	0.006	-0.048	-0.122 ~ 0.025	0.198	0.003	-0.067	-0.157 ~ 0.024	0.150	0.006
rs2049046	-0.039	-0.113 ~ 0.035	0.305	0.002	-0.070	-0.143 ~ 0.004	0.064	0.006	-0.047	-0.138 ~ 0.044	0.314	0.003
rs7934165	-0.038	-0.112 ~ 0.035	0.307	0.002	-0.072	-0.145 ~ 0.002	0.056	0.007	-0.033	-0.124 ~ 0.058	0.480	0.001
rs10767665	-0.033	-0.107 ~ 0.041	0.378	0.001	-0.073	-0.146 ~ 0.001	0.052	0.007	-0.043	-0.134 ~ 0.048	0.351	0.002
rs7931247	-0.026	-0.100 ~ 0.048	0.496	0.001	-0.071	-0.144 ~ 0.003	0.059	0.006	-0.038	-0.129 ~ 0.053	0.416	0.002
rs1491851	0.006	-0.067 ~ 0.080	0.865	<0.001	-0.038	-0.111 ~ 0.036	0.316	0.002	0.077	-0.014 ~ 0.167	0.097	0.007
rs1157659	0.022	-0.052 ~ 0.096	0.555	0.001	-0.045	-0.119 ~ 0.028	0.226	0.003	0.086	-0.004 ~ 0.176	0.061	0.009

Variants	BIS motor (n = 378)				BIS attention (n = 378)				BIS sum (n = 378)			
	beta [a]	95% CI	*p* [b]	partial η^2	beta [a]	95% CI	*p* [b]	partial η^2	beta [a]	95% CI	*p* [b]	partial η^2
rs1519480	-0.031	-0.131 ~ 0.070	0.548	0.001	-0.013	-0.112 ~ 0.086	0.799	<0.001	-0.032	-0.125 ~ 0.061	0.494	0.001
rs6265	-0.033	-0.134 ~ 0.068	0.523	0.001	0.008	-0.090 ~ 0.106	0.874	<0.001	-0.045	-0.138 ~ 0.048	0.341	0.002
rs11030101	-0.029	-0.129 ~ 0.072	0.575	0.001	-0.040	-0.138 ~ 0.059	0.426	0.002	-0.021	-0.114 ~ 0.072	0.655	0.001
rs7940188	-0.029	-0.129 ~ 0.072	0.577	0.001	-0.028	-0.126 ~ 0.071	0.583	0.001	-0.051	-0.144 ~ 0.041	0.278	0.003
rs6484320	-0.064	-0.164 ~ 0.037	0.215	0.004	0.002	-0.098 ~ 0.101	0.975	<0.001	-0.077	-0.170 ~ 0.016	0.104	0.007
rs2049046	-0.006	-0.107 ~ 0.095	0.909	<0.001	0.003	-0.096 ~ 0.102	0.956	<0.001	-0.027	-0.121 ~ 0.066	0.566	0.001
rs7934165	0.001	-0.102 ~ 0.101	0.993	<0.001	-0.003	-0.102 ~ 0.096	0.953	<0.001	-0.017	-0.110 ~ 0.077	0.725	<0.001
rs10767665	-0.006	-0.107 ~ 0.096	0.915	<0.001	-0.001	-0.100 ~ 0.098	0.986	<0.001	-0.027	-0.120 ~ 0.066	0.570	0.001
rs7931247	-0.005	-0.106 ~ 0.097	0.926	<0.001	0.025	-0.074 ~ 0.124	0.619	0.001	-0.023	-0.116 ~ 0.071	0.630	0.001
rs1491851	-0.036	-0.137 ~ 0.065	0.486	0.001	0.038	-0.061 ~ 0.136	0.454	0.002	0.033	-0.060 ~ 0.126	0.485	0.001
rs1157659	-0.023	-0.124 ~ 0.078	0.655	0.001	-0.013	-0.112 ~ 0.086	0.799	<0.001	0.049	-0.044 ~ 0.142	0.298	0.003

Abbreviations: NS: novelty seeking; HA: harm avoidance; BIS: Barratt Impulsiveness Scale; partial η^2 :partial eta-squared

[a] Standardized beta coefficient, representing the mean difference in outcome between minor allele carriers and the reference genotype group.

[b] The total sample with the reference group genotype versus the total sample with variant carriers.

Supplementary Table 2. Association of BDNF gene polymorphisms with specific personality traits and impulsiveness in the control sample using dominant model linear regression, adjusting for age and gender

Variants	NS score (n = 182)				HA score (n = 182)				BIS nonplan (n = 279)			
	beta [a]	95% CI	*p* [b]	partial η^2	beta [a]	95% CI	*p* [b]	partial η^2	beta [a]	95% CI	*p* [b]	partial η^2
rs1519480	-0.069	-0.181 ~ 0.043	0.226	0.005	-0.056	-0.175 ~ 0.064	0.359	0.003	0.064	-0.086 ~ 0.213	0.403	0.004
rs6265	-0.099	-0.211 ~ 0.012	0.080	0.011	-0.015	-0.134 ~ 0.104	0.809	<0.001	0.070	-0.079 ~ 0.218	0.354	0.005
rs11030101	-0.017	-0.129 ~ 0.095	0.764	<0.001	0.054	-0.065 ~ 0.173	0.374	0.003	-0.047	-0.194 ~ 0.101	0.531	0.002
rs7940188	-0.066	-0.178 ~ 0.046	0.248	0.005	-0.055	-0.174 ~ 0.064	0.362	0.003	0.067	-0.081 ~ 0.216	0.373	0.005
rs6484320	-0.110	-0.222 ~ 0.001	0.052	0.014	-0.029	-0.148 ~ 0.090	0.631	0.001	0.050	-0.098 ~ 0.199	0.503	0.003
rs2049046	-0.081	-0.193 ~ 0.030	0.152	0.008	0.001	-0.119 ~ 0.119	0.998	<0.001	0.070	-0.079 ~ 0.218	0.356	0.005
rs7934165	-0.079	-0.190 ~ 0.033	0.166	0.007	-0.015	-0.134 ~ 0.104	0.806	<0.001	0.096	-0.052 ~ 0.244	0.203	0.009
rs10767665	-0.066	-0.178 ~ 0.045	0.244	0.005	-0.018	-0.137 ~ 0.101	0.767	<0.001	0.070	-0.079 ~ 0.218	0.356	0.005
rs7931247	-0.049	-0.160 ~ 0.063	0.394	0.003	-0.030	-0.149 ~ 0.089	0.625	0.001	0.077	-0.071 ~ 0.225	0.304	0.006
rs1491851	0.011	-0.101 ~ 0.124	0.844	<0.001	-0.006	-0.126 ~ 0.114	0.922	<0.001	0.038	-0.109 ~ 0.186	0.609	0.002
rs1157659	0.035	-0.077 ~ 0.148	0.536	0.001	-0.031	-0.151 ~ 0.089	0.615	0.001	0.046	-0.102 ~ 0.193	0.544	0.002

Variants	BIS motor (n = 279)				BIS attention (n = 279)				BIS sum (n = 279)			
	beta [a]	95% CI	*p* [b]	partial η^2	beta [a]	95% CI	*p* [b]	partial η^2	beta [a]	95% CI	*p* [b]	partial η^2
rs1519480	0.057	-0.093 ~ 0.207	0.452	0.003	0.080	-0.068 ~ 0.227	0.289	0.006	0.081	-0.067 ~ 0.229	0.284	0.007
rs6265	0.051	-0.098 ~ 0.199	0.501	0.003	0.081	-0.065 ~ 0.228	0.275	0.007	0.081	-0.066 ~ 0.227	0.279	0.007
rs11030101	-0.011	-0.159 ~ 0.136	0.879	<0.001	-0.024	-0.170 ~ 0.121	0.743	0.001	-0.033	-0.179 ~ 0.113	0.655	0.001
rs7940188	0.050	-0.099 ~ 0.199	0.507	0.003	0.091	-0.055 ~ 0.238	0.220	0.009	0.083	-0.064 ~ 0.230	0.268	0.007
rs6484320	0.063	-0.085 ~ 0.212	0.400	0.004	0.084	-0.062 ~ 0.230	0.259	0.007	0.080	-0.067 ~ 0.226	0.286	0.007
rs2049046	0.065	-0.084 ~ 0.213	0.392	0.004	0.081	-0.066 ~ 0.227	0.278	0.007	0.087	-0.060 ~ 0.234	0.243	0.008
rs7934165	0.080	-0.068 ~ 0.229	0.288	0.006	0.092	-0.054 ~ 0.239	0.217	0.009	0.109	-0.037 ~ 0.256	0.143	0.012
rs10767665	0.065	-0.084 ~ 0.213	0.392	0.004	0.081	-0.066 ~ 0.227	0.278	0.007	0.087	-0.060 ~ 0.234	0.243	0.008
rs7931247	0.071	-0.077 ~ 0.219	0.344	0.005	0.105	-0.041 ~ 0.251	0.157	0.011	0.102	-0.045 ~ 0.248	0.172	0.011
rs1491851	-0.032	-0.180 ~ 0.116	0.668	0.001	0.085	-0.060 ~ 0.230	0.250	0.008	0.029	-0.117 ~ 0.175	0.694	0.001
rs1157659	-0.016	-0.164 ~ 0.132	0.835	<0.001	0.099	-0.046 ~ 0.245	0.179	0.010	0.045	-0.101 ~ 0.191	0.545	0.002

Abbreviations: NS: novelty seeking; HA: harm avoidance; BIS: Barratt Impulsiveness Scale; partial η^2: partial eta-squared

[a] Standardized beta coefficient, representing the mean difference in outcome between minor allele carriers and the reference genotype group.

[b] The control sample with the reference group genotype versus the control sample with variant carriers.
